# Endothelial dysfunction in Marfan syndrome mice is restored by resveratrol

**DOI:** 10.1038/s41598-022-26662-5

**Published:** 2022-12-28

**Authors:** Arnout Mieremet, Miesje van der Stoel, Siyu Li, Evrim Coskun, Tsveta van Krimpen, Stephan Huveneers, Vivian de Waard

**Affiliations:** 1 Department of Medical Biochemistry, Amsterdam UMC Location University of Amsterdam, Meibergdreef 9, Amsterdam, The Netherlands; 2 Amsterdam Cardiovascular Sciences, Atherosclerosis and Ischemic Syndromes, Amsterdam, The Netherlands; 3 Amsterdam Cardiovascular Sciences, Microcirculation, Amsterdam, The Netherlands

**Keywords:** Cardiovascular diseases, Cellular imaging

## Abstract

Patients with Marfan syndrome (MFS) develop thoracic aortic aneurysms as the aorta presents excessive elastin breaks, fibrosis, and vascular smooth muscle cell (vSMC) death due to mutations in the FBN1 gene. Despite elaborate vSMC to aortic endothelial cell (EC) signaling, the contribution of ECs to the development of aortic pathology remains largely unresolved. The aim of this study is to investigate the EC properties in Fbn1^C1041G/+^ MFS mice. Using en face immunofluorescence confocal microscopy, we showed that EC alignment with blood flow was reduced, EC roundness was increased, individual EC surface area was larger, and EC junctional linearity was decreased in aortae of Fbn1^C1041G/+^ MFS mice. This modified EC phenotype was most prominent in the ascending aorta and occurred before aortic dilatation. To reverse EC morphology, we performed treatment with resveratrol. This restored EC blood flow alignment, junctional linearity, phospho-eNOS expression, and improved the structural integrity of the internal elastic lamina of Fbn1^C1041G/+^ mice. In conclusion, these experiments identify the involvement of ECs and underlying internal elastic lamina in MFS aortic pathology, which could act as potential target for future MFS pharmacotherapies.

## Introduction

Marfan Syndrome (MFS) is an autosomal dominant disorder of the connective tissue that is caused by mutations in the FBN1 gene encoding fibrillin-1^1,2^. MFS (OMIM: 154,700) is a systemic disease with an estimated prevalence of ~ 1:10.000^3–5^. The disease is manifested in the cardiovascular, skeletal, and ocular system. Most life-threatening cardiovascular complications are the dilatation of the aortic root and the ascending aorta, which may dissect or rupture^6–8^. To prevent rupture, long-term pharmacotherapies using β-blockers, angiotensin-II receptor blockers or by surgical interventions are performed as described in the medical guidelines^9^.

Fibrillin-1 is an important glycoprotein that forms microfibrils^10,11^. Mutations in the FBN1 gene decrease the availability of fibrillin-1 or alters its structure, interfering with proper microfibril formation^12,13^. Fibrillin-1 acts as a scaffold for elastic fiber formation, although microfibrils also occur independently of elastic fibers^14^. Besides its structural properties in the extracellular matrix (ECM), fibrillin-1 can sequester growth factors to regulate their local availability and activity, indicating the versatility of this protein^11,15,16^.

Extensive ECM-cell interactions are present in the aortic vessel wall that consists of an integrated network of various cell types supported by a durable matrix including elastin fibers and fibrillin-1 microfibrils. Microfibrils tether vascular smooth muscle cells (vSMCs) and endothelial cells (ECs) to the elastic fibers of the ECM^17,18^. Moreover, the Arg-Gly-Asp (RGD) motif of fibrillin-1 binds to a subset of integrins, the main element of cell-ECM adhesions, which regulates cell adhesion^19,20^. The integrin receptors bind to microfibrils that surround the elastin fibers to form a direct link between the ECM and the cellular cytoskeleton. The elastin-contractile units in the vessel wall can directly affect cellular signaling pathways, resulting in changes in cellular behavior such as cell adhesion, shape remodeling, migration, or proliferation^21–23^.

Moreover, the vascular system is constantly subjected to various mechanical forces, such as pulsatile blood flow, cyclic stretching, and vascular stiffening. These mechanical forces subjected on vSMCs and ECs must be balanced by opposite resistant forces, generated by the ECM and actomyosin-mediated contractility, which produces intracellular forces^24,25^. Mechanotransduction of these forces are dependent on cell–cell adherens junctions and cell-ECM adhesions^26^. Upon laminar flow, vSMCs and ECs sense and transduce the mechanical forces into biochemical signaling, thereby changing cellular behavior. Specifically for ECs, this includes alignment in the direction of blood flow and stabilization of adherens junctions^27–29^.

Interestingly, aorta pathology in MFS has been associated with endothelial dysfunction^30–32^. A characteristic of MFS is an increase in aortic stiffness, observed in MFS patients and in MFS mouse models^33–37^. Moreover, patients with MFS have elevated levels of von Willebrand factor and thrombomodulin, which are indicators of endothelial dysfunction^38^. Additionally, patients with MFS have impaired endothelial-dependent vasodilation, which is correlated with aortic aneurysm formation^31,39,40^. However, the effect of mutated FBN1 on EC morphology and the role of ECs during aortic pathogenesis in MFS is still unresolved.

Treatment with resveratrol (RESV) has been shown to exert vasoprotective effects^41^. As we reported earlier, two months RESV treatment halts aortic root dilatation in MFS mice by promoting elastin integrity and vSMC survival in part via its effect on ECs, producing an EC mediator that could downregulate detrimental vSMC-derived miR29b expression^42^. Moreover, RESV enhances endothelial nitric oxide (NO) production through upregulation of phospho-endothelial NO synthase (p-eNOS) expression and its subcellular localization on cell–cell contacts^43,44^. Yet, the direct effects of RESV on ECs, especially focusing on the morphological changes, remained unresolved.

In this study, we aim to elucidate the EC organization in a MFS mouse model. By using *en face* immunofluorescence (IF) visualization of ECs in the aorta of the *Fbn1*^C1041G/+^ MFS mouse model, we revealed an altered EC morphology in the thoracic ascending and descending aorta. Moreover, we describe the efficacy of RESV treatment on the normalization of EC morphology and number of elastin breaks in the aorta. The findings provide novel insights on the contribution of ECs to the aortic pathology in MFS and pinpoint the endothelial tissue as a potential novel therapeutic target for this rare disease.

## Results

### Morphological changes in aortic endothelial cells of MFS Fbn1^C1041G/+^ mice

**Figure 1:**
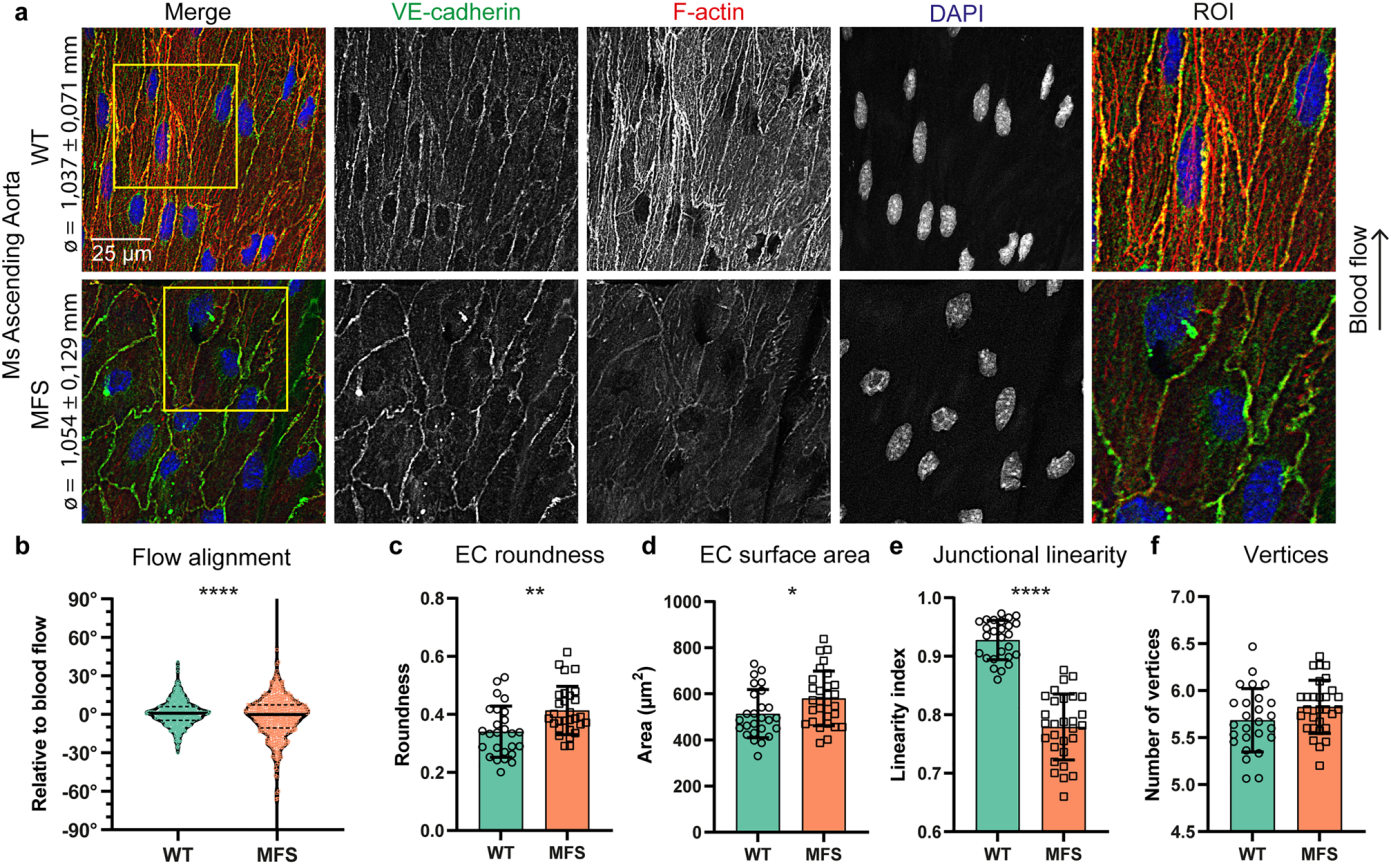
MFS ECs have altered morphological characteristics in the pre-dilated ascending aorta. (**a**) Representative *en face* IF confocal images showing VE-cadherin (green), F-actin (red), and DAPI (blue) of ascending aorta from 9 weeks old Fbn1^C1041G/+^ MFS mice and WT littermates indicated with aortic diameter. Region of interest (ROI) highlights EC junctional linearity. Arrow indicates the direction of blood flow. Scale bar: 25 μm. (**b**) Violin plot showing quantification of EC alignment normalized to the direction of flow. Data distribution is tested by Kolmogorov–Smirnov test, ****p < 0.001. (**c**–**f**) Bar plots of EC morphology parameters in the ascending aorta from 9 weeks old Fbn1^C1041G/+^ MFS mice and WT littermates. All data represents mean ± SD with indicated datapoints for WT (N = 6; M/F = 3/3) n = 25 images (total 424 ECs) and MFS (N = 6; M/F = 4/2) n = 33 images (total 555 ECs), *p < 0.05, **p < 0.01, ****p < 0.0001 analyzed by students t-test.

We assessed the morphology of ECs in the ascending aorta, the location with frequent aortic aneurysms and dissections in MFS. To this end, transmembrane protein complexes of ECs were visualized by *en face* visualization of either VE-cadherin or β-catenin, which are both part of the same cell–cell junctional adhesion complex. In the outer curvature of the ascending aorta in 9 weeks old wild type (WT) mice, ECs were aligned with the direction of blood flow, characterized by elongated ECs, with the nucleus at the downstream side, and with straight cell–cell junctions (Fig. 1a). In contrast, the ECs in the outer curvature of the ascending aorta in 9 weeks old MFS Fbn1^C1041G/+^ mice appeared less elongated, more round in shape, with the nucleus more in the center of the EC, and not aligned with the blood flow as compared to WT (Fig. 1a). Aortic diameters were similar, indicating a pre-dilated state for the MFS mice. To methodologically assess morphological features, EC shapes were quantified based on five different parameters: alignment with flow, cell roundness, cell surface area, junctional linearity (i.e. junctional stability), and number of vertices (methodology illustrated in Fig. S1). This demonstrated that ECs in MFS mice show a substantial reduced alignment with flow, increased roundness of the cells, an enlarged cell surface area, a reduced junctional linearity index, while the number of vertices was similar between genotypes (Fig. 1b–f). The analysis of these parameters show that the ECs in the MFS ascending aorta demonstrate strong organizational changes that are present before dilatation occurs.

### Milder effect of mutated FBN1 on EC morphology in thoracic descending aorta

**Figure 2:**
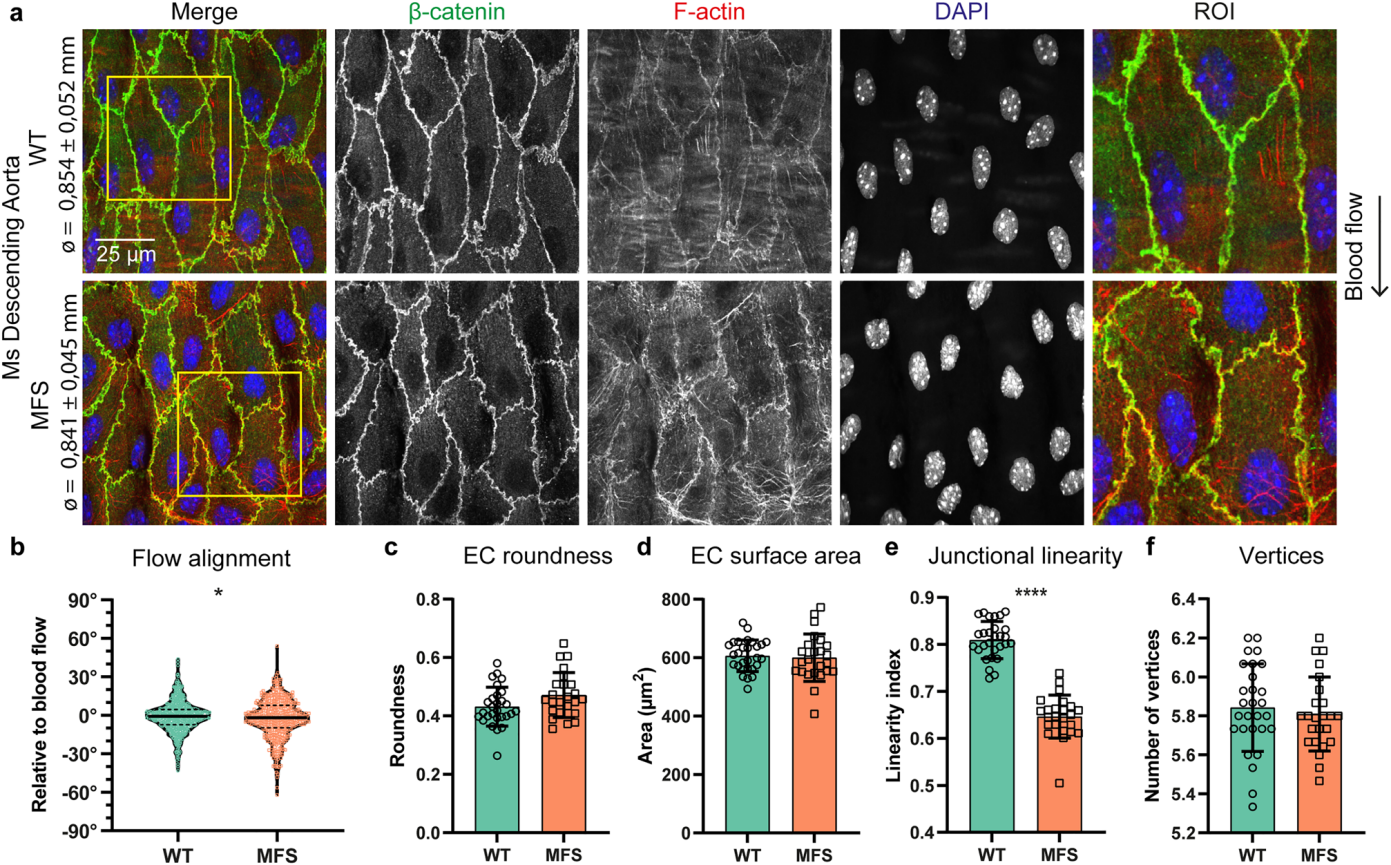
Reduced flow alignment and junctional linearity in ECs of the non-dilated thoracic descending aorta. (**a**) Representative *en face* IF confocal images showing β-catenin (green), F-actin (red), and DAPI (blue) of thoracic descending aorta from 9 weeks old *Fbn1*^C1041G/+^ MFS mice and their WT littermates indicated with aortic diameter. EC junctional shape is highlighted in the ROI, and direction of blood flow is indicated with an arrow. Scale bar: 25 μm. (**b**) Violin plot showing quantification of EC alignment normalized to the direction of flow. Data distribution was analyzed by Kolmogorov–Smirnov test, * indicates *p* < 0.05. (**c**–**f**) Bar plots of EC morphology parameters in the ascending aorta from 9 weeks old *Fbn1*^C1041G/+^ MFS mice and WT littermates. All data are represented as mean ± SD with indicated datapoints for WT (N = 7; M/F = 3/4) n = 28 images (total 420 ECs) and MFS (N = 6; M/F = 2/4) n = 24 images (total 360 ECs). ****p < 0.0001 analyzed by students t-test.

We further assessed EC morphology in the thoracic descending aorta of the MFS mice. This region is not prone to aneurysm formation or dissections in this rodent model. As compared to WT and MFS ascending aorta (Fig. 1a), ECs from their respective genotypes were substantially less elongated and more round in shape in the descending aorta, probably attributed to the lower shear stress (Fig. S2). Comparing the descending aortae only, the aortic diameters were similar between WT and MFS as expected in this model (Fig. 2a). In the three-dimensional representation of the *en face* staining, a clear separation of the EC and vSMC layer can be observed (Movies S1, S2). Notably, the orientation of the vSMCs is perpendicular to the orientation of the ECs. The e*n face* images showed subtle differences in the morphology of ECs between WT and MFS (Fig. 2a). Quantification of the EC morphology was performed using the five shape parameters (Fig. 2b–f). This revealed a substantial difference in the alignment of the ECs with the blood flow (Fig. 2b) and a substantial decrease in junctional linearity index (Fig. 2e). The latter was best observed in the ROI of the images, as EC junctions were straight in WT and jagged in MFS. Shape quantification in WT and MFS showed comparable cell roundness, surface area, and number of vertices. Taken together, endothelial disorganization is evident early in pathogenesis of MFS and particularly demarcates aneurysm-prone aortic locations.

### EC alignment and junctional linearity aberrations in MFS ascending aorta ameliorated by resveratrol treatment

Subsequently, our experiments focused on the restoration of the EC organization in the aorta of the MFS model. Therefore, we therapeutically targeted ECs by administration of the polyphenol RESV in drinking water during three weeks to 36 weeks old *Fbn1*^C1041G/+^ MFS mice, which is at an age where the aortic pathology has been aggravated^45^.

Initially, the histology of the aorta in 36 weeks old *Fbn1*^C1041G/+^ MFS mice treated with either vehicle (MFS-CTRL) or RESV (MFS-RESV) was compared to untreated WT littermates. As expected at this age, the aortic root showed aneurysmal dilatation at 36 weeks in female *Fbn1*^C1041G/+^ MFS mice as compared to age and sex matched WT controls (Fig. S3A,B). The three week treatment with RESV in MFS mice did not change the enlarged diameter of the aortic root. The ascending aorta of MFS-CTRL and MFS-RESV mice showed no dilatation as compared to WT controls (Fig. S3C,D), which is common in this mild MFS model.

When focusing on the effect of RESV treatment on the EC morphology in the ascending aorta, we visualized EC adherens junctions using the *en face* IF method in 36 weeks old vehicle and RESV treated *Fbn1*^C1041G/+^ MFS mice. As these were older than the 9 weeks old mice used in the previous experiments, we first compared EC morphology between the 9 weeks old MFS mice with the 36 weeks old *Fbn1*^C1041G/+^ MFS mice. Due to ageing, we observed a further decline in junctional linearity and a reduction in cell surface area in the 36 weeks old MFS mice as compared to the 9 weeks old MFS mice (Fig. S4). The EC alignment with blood flow, number of vertices, and roundness remained similar during the pathogenic process (Fig. S4).

**Figure 3:**
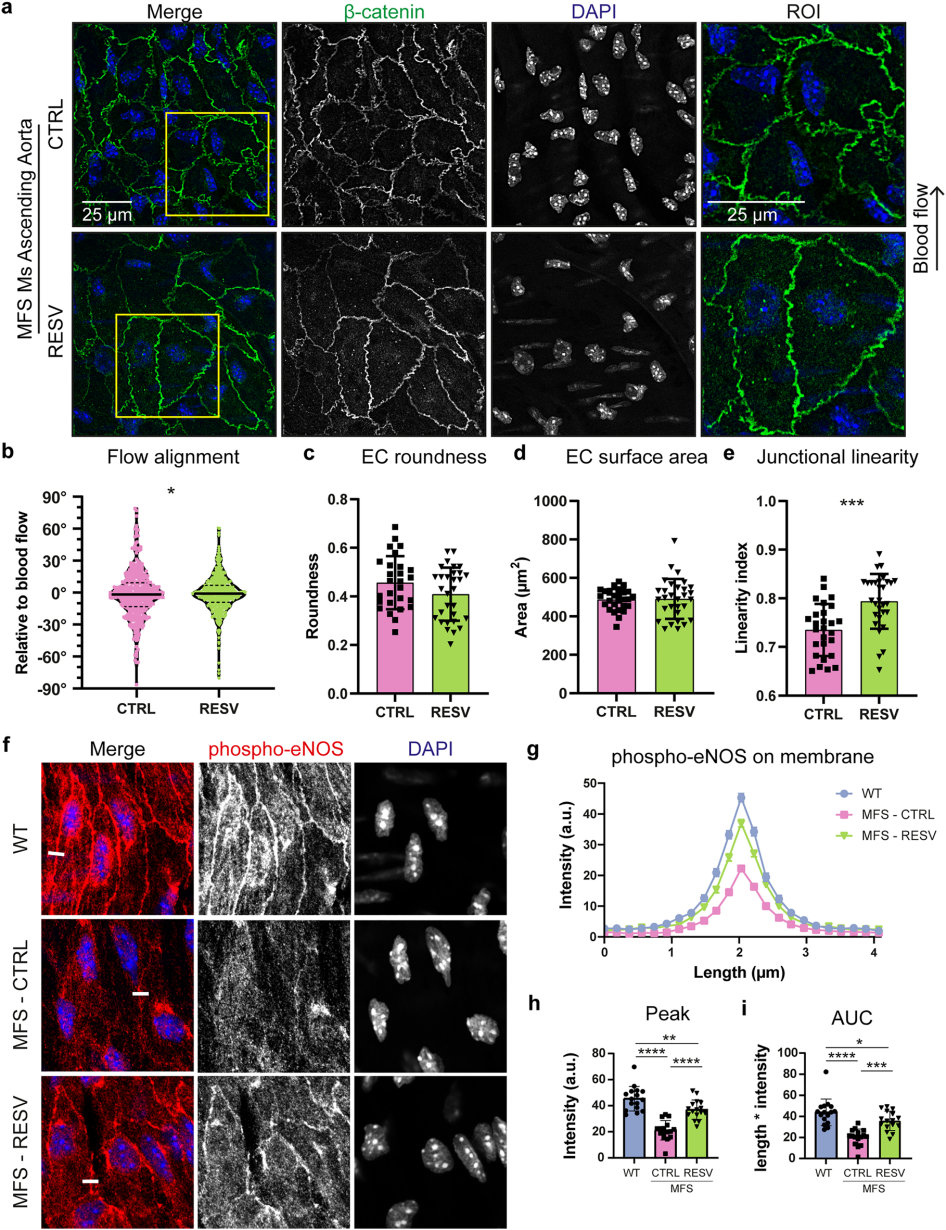
Normalization of EC morphology after resveratrol treatment in the ascending aorta in MFS mice. (**a**) Representative *en face* IF confocal images showing β-catenin (green), and DAPI (blue) of ascending aorta from 36 weeks old *Fbn1*^C1041G/+^ MFS mice treated for 3 weeks with either vehicle control or RESV. Region of interest (ROI) highlights EC junctional linearity. Arrow indicates the direction of blood flow. Scale bar: 25 μm. (**b**) Violin plot showing quantification of EC alignment normalized to the direction of flow. Data distribution was analyzed by Kolmogorov–Smirnov test, * indicates *p* < 0.05. (**c**–**e**) Bar plots of EC morphology parameters in the ascending aorta from 36 weeks old *Fbn1*^C1041G/+^ MFS mice treated for 3 weeks with either vehicle control or RESV. All data are represented as mean ± SD with indicated datapoints for vehicle treatment (N = 6; M/F 0/6) n = 24 images (total 360 ECs) and RESV treatment (N = 6; M/F 0/6) n = 24 images (total 360 ECs). (**f**) Representative *en face* IF confocal images showing p-eNOS (Ser1177) (red) and DAPI (blue) of ascending aorta from 36 weeks old WT, *Fbn1*^C1041G/+^ MFS mice treated for 3 weeks with either vehicle control or RESV. Line of quantification indicated as white line. (**g**) Line plot of p-eNOS quantification over a length of 4 µm perpendicular over the EC membrane in WT, MFS-CTRL, and MFS-RESV. (**h**, **i**) Bar plots comparing peak intensity and area under the curve (AUC) of p-eNOS. Data represents mean mean ± SD with indicated datapoints for WT (N = 4; M/F 0/4) n = 16 images (total 80 ECs), vehicle treatment (N = 4; M/F 0/4) n = 16 images (total 80 ECs), and RESV treatment ((N = 4; M/F 0/4) n = 16 images (total 80 ECs). *p < 0.05, **p < 0.01, ***p < 0.001, ****p < 0.0001 analyzed by students t-test or one-way ANOVA with Holm-Sidak post-test.

Next, we analyzed the effect of RESV treatment on the endothelium by comparing 36 weeks old MFS-CTRL with MFS-RESV. In the ascending aorta of vehicle treated *Fbn1*^C1041G/+^ MFS mice, ECs were round and not clearly aligned with the flow, where this seemed improved upon RESV treatment (Fig. 3a). Moreover, the junctions were more jagged in the MFS-CTRL, whereas junctions were more linear in the MFS-RESV. Quantification revealed that RESV indeed improved EC alignment with flow and increased the junctional linearity index (Fig. 3B,E). The roundness, cell surface area (Fig. 3C,D), and number of vertices (MFS-CTRL = 5.80 ± 0.35 vs MFS-RESV = 5.65 ± 0.23) remained unaltered.

As part of the RESV effect are mediated by eNOS^46^, we evaluated the expression of phosphorylated eNOS (p-eNOS) at serine 1177 in the ascending aorta. Specifically, we evaluated the expression of p-eNOS at the membrane junctions, as eNOS targeted to the plasma membrane generates the most NO^47^. In the ascending aorta of WT, higher levels of p-eNOS were present at cell–cell connections as compared to MFS-CTRL (Fig. 3f). Treatment with RESV reintroduced p-eNOS at the EC membrane. Quantification of p-eNOS on the cellular membrane showed restoration of p-eNOS fluorescence intensity in MFS-RESV for peak height and area under the curve (AUC), more closely resembling WT (Fig. 3g–i).

In addition, the EC morphology in the thoracic descending aorta of 36 weeks old *Fbn1*^C1041G/+^ MFS mice with RESV treatment was evaluated. Quantification of the EC morphological parameters revealed no substantial differences after RESV treatment in the descending aorta (Fig. S5). Therefore, the beneficial effects of RESV are mainly present in the disorganized EC features located in the ascending aorta.

### Intercellular matrix alterations in MFS were improved by resveratrol treatment

A hallmark for thoracic aortic aneurysm formation in MFS is the high level of elastic lamina breaks. Therefore, we investigated the elastic lamina breaks using two methods. With a Lawson staining of aortic cross sections, the number of elastin breaks throughout the medial layer was determined. Complementary, with an *en face* approach the fraction of disrupted elastin in the internal elastic lamina (IEL), which forms the direct connection between the aortic intima (i.e. ECs) and media (i.e. vSMCs), was quantified.

**Figure 4:**
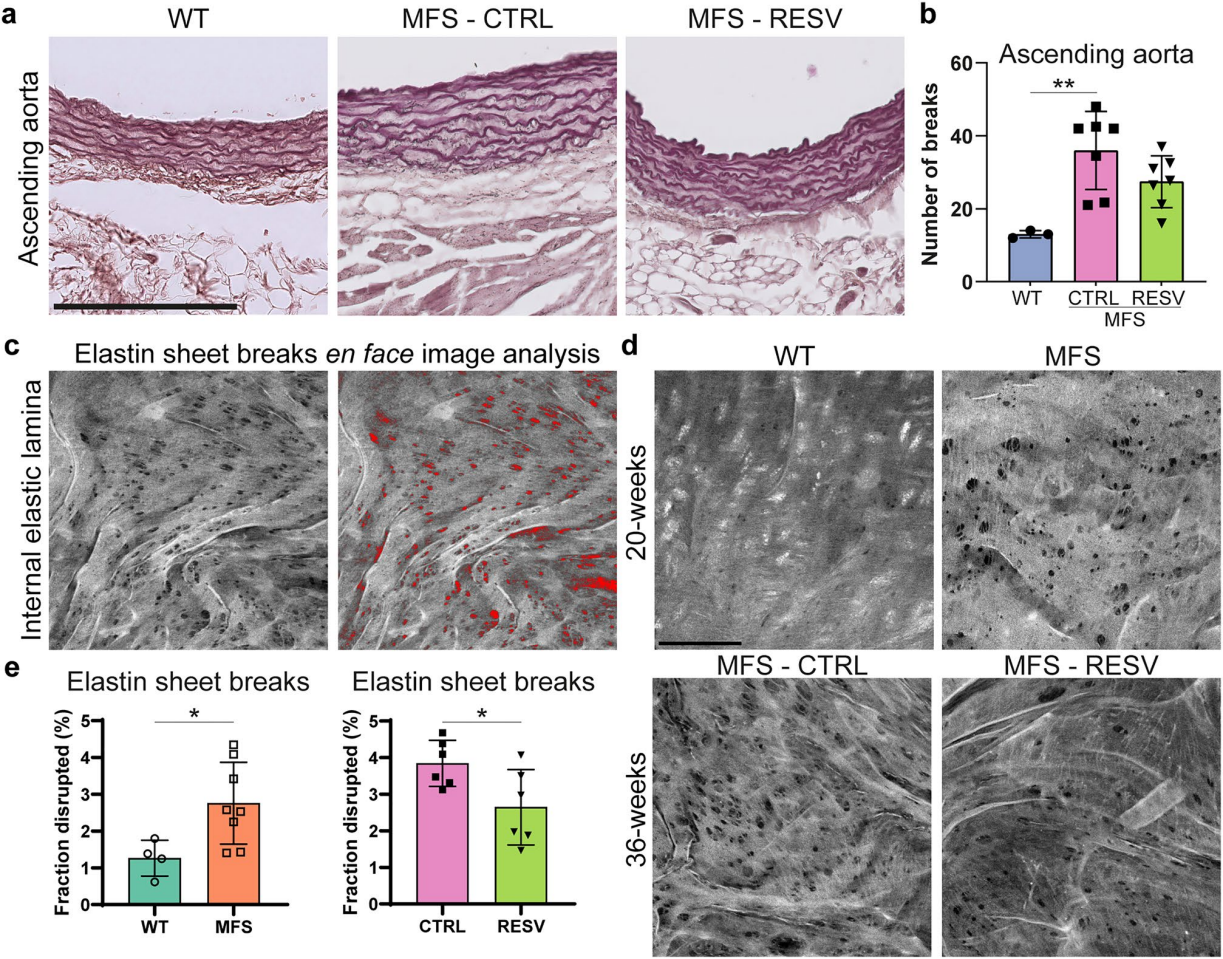
Elastin sheet disruptions in MFS mice are restored by resveratrol treatment. (**a**) Representative images of the elastin lamellae in ascending aorta sections visible after Lawson staining. Scale bar indicates 200 µm. (**b**) Bar plot comparing the number of elastin fiber breaks in the ascending aorta of 36 week old mice. WT (N = 3; M/F 0/3) n = 9 images, MFS-CTRL (N = 7; M/F 0/7) n = 21 images, and MFS-RESV (N = 7 M/F 0/7) n = 21 images. (**c**) Image analysis of autofluorescent internal elastic lamina (IEL) captured by *en face* confocal imaging. Wavy elastin lamellae with fenestrae are shown in maximal projection. Quantification of the disrupted area (in red) is performed and presented as percentage of the total elastin area. (**d**) Representative images of IEL in 20 weeks old WT and Fbn1^C1041G/+^ MFS mice in top panel, and of 36 weeks MFS-CTRL and MFS-RESV in the bottom panel. Scale bar indicates 50 µm (**e**) Bar plot comparing the fraction of disrupted IEL in WT WT (N = 4; M/F 0/4) n = 12 images versus MFS (N = 8; M/F 0/8) n = 25 images (left) and MFS-CTRL (N = 6 M/F 0/6) n = 23 images versus MFS-RESV (N = 6 M/F 0/6) n = 22 images (right). All quantified data represents mean ± SD with indicated data points.

To determine the number of elastin breaks in aortic cross sections, the Lawson staining was performed to reveal the elastic lamellae in the ascending aorta (Fig. 4a). In this aortic region, a substantial higher number of breaks in *Fbn1*^C1041G/+^ MFS mice was observed as compared to WT (Fig. 4b). After 3 weeks of RESV treatment, the number of elastin breaks in the aortic media of the ascending aorta in the MFS mice was not significantly reduced (*p* = 0.16).

Visualization of the IEL, which is the most luminal elastin sheet closest to the ECs, was performed based on the autofluorescent nature of the elastin protein. Data was acquired using an *en face* z-stack collection of confocal microscopy images capturing only the IEL. Subsequently, the maximal projection of the z-stack collection revealed the presence of disruptions (i.e. fenestrae) in the IEL. These were measured using a custom developed analysis pipeline in ImageJ software to quantify the level of elastin sheet disruptions (Fig. 4c). The total level of disrupted elastin was increased in the MFS mice as compared to WT (Fig. 4d,e). When focusing on the effect of RESV treatment on the disruption of the IEL, 36 weeks old MFS-CTRL were compared to MFS-RESV. This showed that the 3 week RESV treatment in MFS mice reduced the fraction of disrupted IEL (Fig. 4d,e), which indicates a partial restoration of the IEL integrity after RESV treatment. Potentially, the improved EC phenotype after RESV treatment is attributed to the combination of improved p-eNOS signaling and underlying elastin sheet repair that is achieved locally.

## Discussion

**Figure 5:**
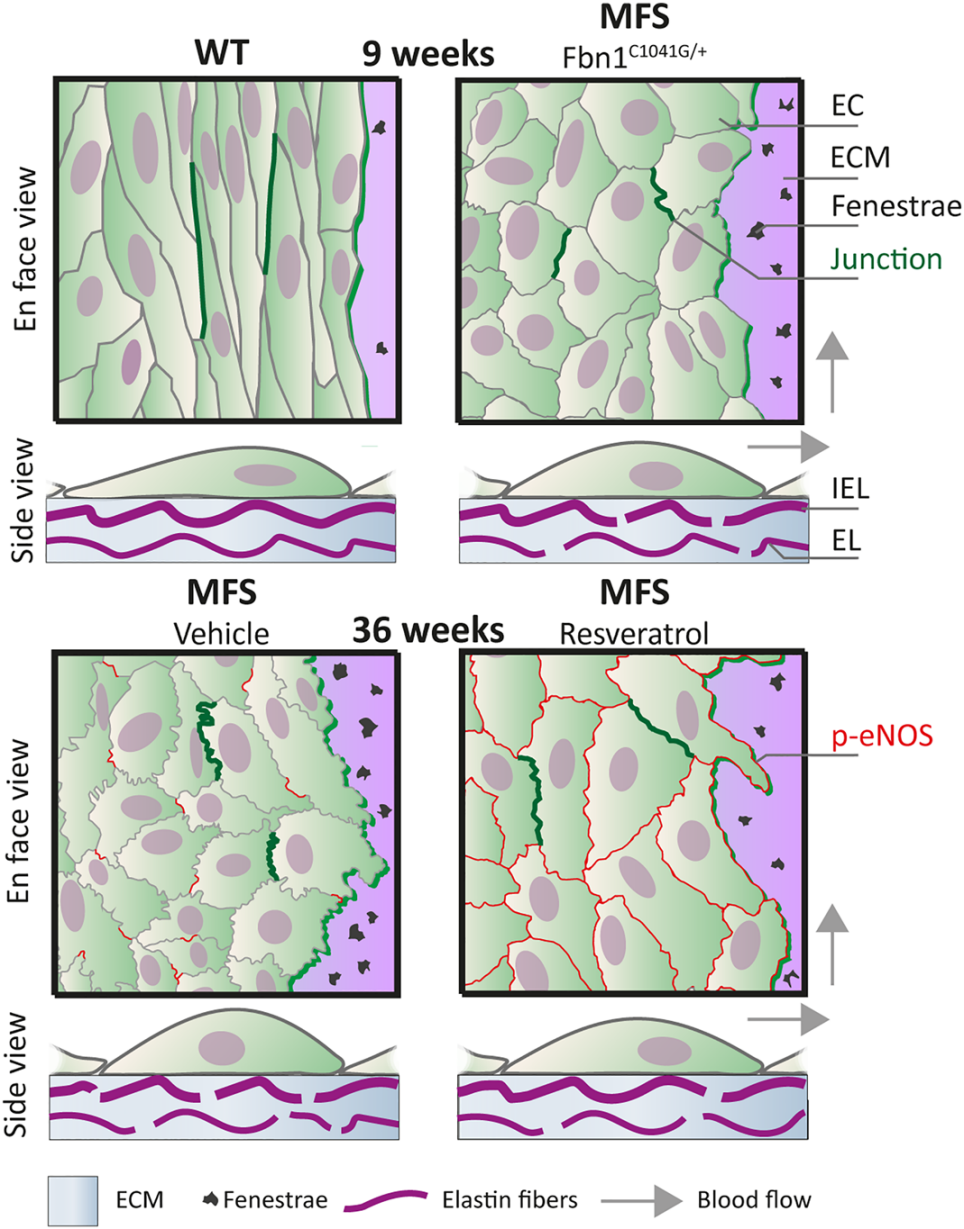
Overview of endothelial cell dysfunction and elastin sheet disruptions in a mouse model of MFS. Schematic presentation of endothelial cell (EC) organization and extracellular matrix (ECM) structure in the thoracic ascending aorta of WT, MFS, MFS-CTRL, and MFS-RESV mice. In the en face view, differences in EC morphology are shown and two adherens junctions connecting ECs are indicated with a dark green line to highlight the junctional linearity. Moreover, the elastin sheet on which the ECs are situated is drawn with occasional fenestrae. Expression of p-eNOS on the EC membrane is shown as red lines at the age of 36 weeks. In the side view, EC morphology is shown, and the fenestrae are now indicated as breaks in the internal elastic lamina (IEL). Part of the aortic media is shown with its ECM and a medial elastic lamina (EL) (vSMCs are not depicted). Arrows indicate blood flow direction.

In summary, these results show that the morphology of the ECs within the aorta of a representative model for MFS is altered and reveal that ECs may act as important mediator in the development of aorta pathology in MFS (Fig. 5). Specifically, EC alignment with flow, roundness, cell surface area, and junctional linearity were changed in the ascending aorta of MFS mice, when compared to age-matched WT littermates with similar aortic dimensions. This revealed for the first time that these endothelial changes occur prior to dilatation, suggesting a contributing role. In the thoracic descending aorta, which hardly shows aortic pathology in this MFS model, only the EC flow alignment and junctional linearity were reduced in MFS mice. This indicates that EC morphological alterations are more severe at the location where aortic aneurysms mostly occur, which is also the location subjected to enhanced mechanical forces.

The ECs emerged as target for RESV in our previous study, as RESV reduced aortic dilatation in an EC-dependent manner by decreasing the expression of aneurysm formation associated miR-29b in vSMCs^42,48^. Here, we attempt to unravel what effect RESV could have on the dysfunctional EC layer in the MFS aorta. Our data shows that RESV reversed the disturbed EC flow alignment, junctional linearity, p-eNOS expression, and integrity of the elastin sheet directly underneath the EC layer. Since fibrillin-1 is the core of elastin fibers and enhanced disruptions in the IEL adjacent to the endothelium were repaired upon RESV in MFS, this could be a direct consequence of the improved EC phenotype after RESV treatment. Potentially, reduced leakage of factors from the circulation or from dysfunctional ECs due to reduced elastin disruptions upon RESV, may also have a beneficial effect on the vSMC in the media. Our study extends current knowledge on aorta pathology in MFS and highlights the potential of targeting EC pathology to promote aortic health in MFS.

Recent clinical measurements on aortic blood flow dynamics in MFS patients revealed different flow patterns in the ascending aorta^49–51^. As the flow-derived forces (i.e. shear stress and circumferential stretch) have been shown to induce dynamic changes in EC shape and polarity^52,53^, this will lead to sustained and potentially progressive alterations in EC morphology. Functionally, impairment of flow-mediated EC-dependent vasodilation was described in MFS patients, which was correlated to aortic dilatation^31^, and can be attributed to disturbed anchoring of ECs to the subendothelial matrix^54^. Our data showed that the altered EC morphology was already observed before dilatation occurred, also suggesting that the effect of mutated FBN1 was perceived by ECs most probably due to alterations in the structure or composition of the intimal basement membrane and IEL. Our findings on the more disrupted IEL in the MFS model are in line with observation of López-Guimet et al*.*^55^, who also showed these disruptions in IEL structure by *en face* multiphoton microscopy. An increase in number and size of these fenestrae in the IEL has been linked to aortic aneurysm formation in mouse models^55,56^. The higher level of disruption in the IEL facilitates enhanced exchange of paracrine factors between the aortic intima and media through these fenestrae and enables more direct EC to vSMC contact^57^.

A combination of altered flow-derived forces and structural alterations in the ECM in MFS are translated into modified mechanoresponses in ECs. This modulates EC organization, EC shape, nuclear shape, and intracellular organelle positioning^58,59^. A recent study by Zhang et al*.*^60^ linked the formation of aortic aneurysms to defective mechanotransduction in ECs, due to distorted nuclear shape and positioning, and reduced EC flow alignment. An ECM-derived aspect participating in altered mechanoresponse is transforming growth factor-β (TGF-β), as fibrillin-1 plays a key role in sequestration of extracellular TGF-β, the EC-vSMC paracrine communication in MFS may involve changes for this growth factor^61^. As prolonged TGF-β signaling has been associated with endothelial dysfunction^62^, this could be as such partly responsible for the modified EC phenotype as well.

In the murine MFS model, we showed impaired EC alignment with flow in the ascending aorta and in the descending aorta, which is a morphological alteration similar as observed during disturbed flow at bifurcations and branch points throughout the vasculature^63,64^. In the descending aorta, the ECs appeared less stretched-out as compared to the ascending aorta, in line with previous in vitro observations due to lower shear stress^65,66^. Moreover, the reduced junctional linearity showed largest differences between WT and MFS, which has been observed in new forming or remodeling adherens junctions^67,68^. The linear junctions in WT ECs are considered as matured, show enhanced stability, and are attached to actin fibers that are oriented parallel to the cell–cell connection. The jagged junctions of MFS ECs are considered as immature, and connect to intracellular actin cytoskeletal stress fibers positioned mostly perpendicular to the cell membrane^67,69^. Impaired cell–cell junction linearization has been observed during development in a zebrafish model with induced compromised focal adhesion function, thereby linking defective cell-ECM to immature cell–cell connections^70^*.* Treatment with RESV induced partial preservation of EC alignment with flow, junctional linearity, and p-eNOS expression, which could also be linked to improved EC-ECM and EC-EC contacts.

Previously we showed that RESV reduced the aortic pathology in MFS mice^42^, and in this study we have provided more in depth understanding of the beneficial role that RESV can play to improve EC dysfunction and elastin structure. As RESV is a multi-target compound with many different mechanisms of actions^71–74^, not all pathways have been explored in this study to confirm direct or indirect targeting by RESV leading to improved p-eNOS and reduced elastin breaks. In previous research, the RESV effect on ECs and vSMCs are often referred as enhancing energy metabolism by upregulating sirtuin-1 and AMP-activated protein kinase activation^75^, reducing oxidative stress by NADPH oxidases^76^, upregulating endothelial NO production^46^, and reducing adherence of circulating monocytes^77^. While it has been shown that increased mitochondrial fitness and reducing NO-mediated stress decreases MFS aorta pathology^78,79^, yet the question remains how RESV, or one of its metabolites, accomplishes all the above effects and influences the various pathways.

Targeting the NO signaling pathway in MFS aortae has been shown to modulate aneurysm formation, although NOS isoform specific effects were observed^80–82^. When focusing on eNOS, it localizes at plasma membrane caveolae^83^, which is regulated by flow sensing^84^, therefore it seems that RESV also contributes to restoration of the mechanosensing through the caveolae in ECs^85^. Moreover, mechanosensing is associated with the YAP/TAZ transcription factor signaling pathway, which indicates that the effect of RESV on mechanosensing signaling pathways in MFS ECs can be further explored in future research.

MFS patients are often prescribed angiotensin-II receptor blockers (ARBs), of which losartan is most commonly used. Interestingly, ARBs have also been shown to have an angiotensin-II receptor independent protective function involving enhancement of EC function^32,86,87^, although treatment effects are diverse over the various FBN1 genotypes^88^ and for the various ARBs^89^. Moreover, ARBs were effective in reducing aneurysm formation in angiotensin-II receptor deficient mice by targeting EC function^32^. Since ARBs have been shown protective in MFS patients in the meta-analysis of most ARB clinical trials, a potential contributing mechanism is through modulation of ECs^90^. These data show that ECs may be a suitable target cell type in MFS aortic disease, since ECs are more readily in contact with circulating compounds than vSMCs.

In summary, our findings show that ECs in the pre-dilated aorta have morphological alterations in MFS mice, that were aggravated upon age. ECs act as target for RESV to restore EC morphology, p-eNOS expression, and aortic elastin integrity. In future work, the effects of RESV in a human MFS patient population should provide more insight on its effects on aortic functionality to combat cardiovascular complications^91^.

## Methods

### Animal models

Mice were maintained in accordance with the guidelines of the Amsterdam UMC location Academic Medical Center and the Directive 2010/63/EU of the European Parliament. Animal care and experimental procedures were approved by the independent animal experimental committee for Animal Welfare of the Academic Medical Center in Amsterdam. Fbn1^C1041G/+^ MFS mice on a C57Bl6 background were generated from a heterozygous breeding colony. Both male and female mice were included in this study on protocols DBC53AH-1, DBC18-4608-1-2 and -4. All experiments were in compliance with the ARRIVE guidelines. Details regarding the age of mice used in experiments are given in the description of results. Mice were anesthetized and euthanized by a single intraperitoneal injection of a cocktail containing 240 mg/kg ketamine and 24 mg/kg xylazine. The euthanized mice were exsanguinated and perfusion-fixed followed by collection of heart tissue and the thoracic aorta. The collected thoracic aortae contain the ascending aorta, arch, and proximal thoracic descending aorta. Tissues were fixed with 4% PFA in phosphate buffered saline (PBS) enriched with 1 mM CaCl_2_ and 0.5 mM MgCl_2_ (PBS++) and stored at 4 °C before further processing.

### *En face* immunofluorescence staining and image acquisition

A small ring of the aorta was cut using precision scissors and tweezers (Fine Science Tools, Heidelberg, Germany) under a stereo microscope. The tissue ring was then cut open at the inner curvature. Subsequently, the rectangular aorta tissue was stretched and pinned down on a gel-coated petridish using small pins with the endothelium faced upwards. The aorta tissue was washed 3 times with PBS++ before permeabilization in 0.1% Triton-X in PBS++ for 15 min, followed by blocking of the tissue with a 2% bovine serum albumin (BSA) in PBS++ solution for 30 min. Primary antibodies were diluted in Pierce Immunostain Enhancer Solution (ThermoFisher) and incubation of the aorta tissue occurred for 1 h at room temperature. The following primary antibodies were used: polyclonal anti-VE-cadherin (#160840, Cayman Chemicals, Ann Arbor, MI, USA), mouse anti-β-Catenin (#610153, BD Bioscience, Franklin Lakes, NJ, USA), or rabbit anti-Phospho-eNOS (Ser1177) (#9570S, Cell signaling technology Danvers, MA, United States). Tissue was washed three times with PBST++ (PBS++ enriched with 0.1% Tween-20) followed by a 30 min incubation with 4’,6-diamidino-2-phenylindole (DAPI). Afterwards, Alexa-488 or Alexa-568 conjugated secondary antibodies (ThermoFisher) were diluted in Pierce Immunostain Enhancer Solution and tissue was incubated for 1 h. Optionally, phalloidin conjugated to Alexa-488 or Alexa-568 fluorophore (ThermoFisher) was added to the secondary antibody mix. After incubation, tissue was washed 3 times in PBST++ followed by a single wash step in H_2_O. To mount the tissue, pins were removed under a stereo microscope and the aortic tissue was stretched with the endothelium facing the cover glass. Tissue was mounted in Mowiol4-88/DABCO solution overnight with some light weights placed on top of the microscope glass to keep the tissue flattened. Images were acquired under a Leica TCS SP8 X coupled to a Leica DMI6000 confocal microscope equipped with a 63x/1.40 Oil CS2 objective. Determination of the aorta diameter was performed on the stretched-out aortic tissue samples after brightfield imaging.

### Endothelial cell morphology and protein expression analysis

For all morphological parameters (Fig. S1), confocal images were analyzed using ImageJ software (National Institute of Health)^92^. Cell surface area and roundness were measured manually using the freehand selection tool with set measurements for area, perimeter and shape descriptors. Number of vertices was counted manually. The total length of straight lines between vertices was divided by the perimeter to obtain the junctional linearity index for a single EC. For each biological sample, at least four different images were analyzed. In every image at least 15 ECs were randomly selected for quantification. Quantification of p-eNOS on the cell–cell junction was performed using Leica Application Suite X software. Quantification was performed using a line segment with a length of at least 4 µm perpendicular over the cell junction. Background values were subtracted and normalized values were used to calculate peak height and AUC using Graphpad Prism 9.1 (La Jolla, CA, USA).

### Histochemical staining and quantification

Aortic tissue was cut parallel to the aortic root before dehydration and embedding in paraffin to obtain optimal cross sections of the root and ascending aorta as described before^93^. Tissue slices were cut as 7 µm thick sections using a Microm HM340E microtome (ThermoFisher). After deparaffinization and rehydration, the hematoxylin and eosin (HE) staining or Lawson’s elastin staining were performed according to manufacturer’s procedure (Klinipath). Sections were imaged on a Leica DM6B light microscope. Measurements of the aorta root and ascending aorta diameter were performed on three different sections for each sample using the measure tool of Leica Application Suite X software (Leica). Number of elastin breaks was determined by manually counting the locations that showed a break in elastin fibers on a complete preparation. Three different sections for each sample were assessed by two independent blinded observers.

### Internal elastic lamina disruption quantification

Determination of the fraction of disruption in the internal elastic lamina was performed using an *en face* method by imaging the autofluorescence signal of elastin fibers. Nuclei were counterstained with DAPI to determine the location within the sample. Images were acquired with a Leica TCS SP8 X confocal microscope coupled to a Leica DMI6000 camera. Specifically, only the first elastin sheet, which is located between the ECs of the intima and the first vSMC layer of the media, was imaged in z-stack mode and presented as maximum projection. Quantification of the data was performed using ImageJ software (National Institute of Health).

### Statistical analyses

All data was processed and tested for significance using Graphpad Prism 9.1. All data is presented as mean ± SD, and number of ECs that were quantified described for each experiment. Threshold for significant difference was set at *p* < 0.05. In general, student’s t-test was used to compare the means of two groups and 1-way ANOVA was used to compare the means of more than two groups. Data distribution for EC flow alignment was tested by the Kolmogorov–Smirnov test.

## Supplementary Information

Supplementary Information 1.

Supplementary Video 1.

Supplementary Video 2.

## Data Availability

The raw data obtained during this study and processed data used for analysis is available upon reasonable request from the corresponding author.
