# Supplementary material for: Endothelial dysfunction in Marfan syndrome mice is restored by resveratrol
Source: Sci Rep. 2022 Dec 28;12:22504. doi: 10.1038/s41598-022-26662-5 (PMC9797556; doi:10.1038/s41598-022-26662-5)
Supplement: Supplementary file 1 — Supplementary Information 1. [file 41598_2022_26662_MOESM1_ESM.docx]

**Supplementary Information**

**
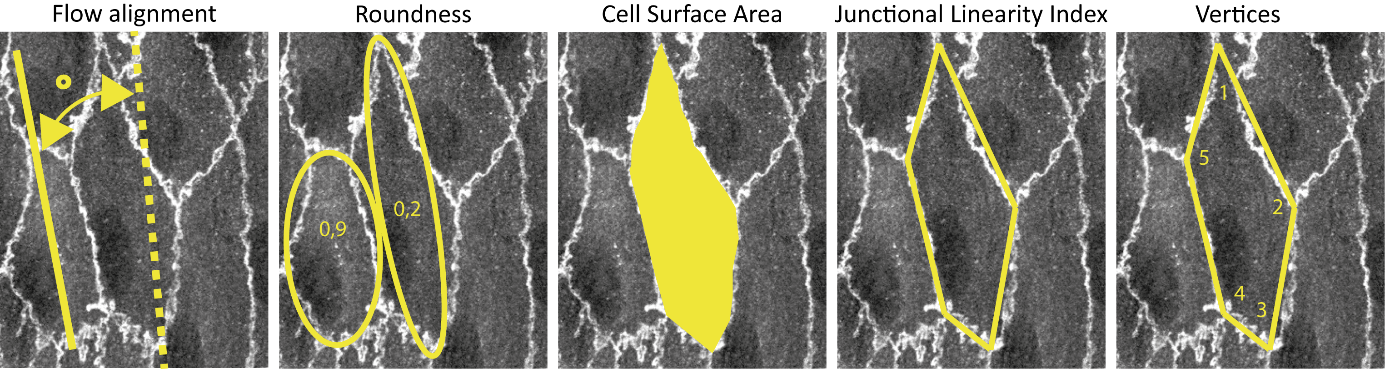
**

**Supplementary figure 1. Endothelial cell shape quantification method analyzing five morphological parameters.**

Alignment with blood flow is calculated for single ECs by measuring the angle between a line connecting two most opposite points of the cell calculated relative to the direction of the flow. Roundness is determined for each cell, for which a more round shape results in a value closer to 1, whereas a more elongated shape results in a roundness value closer to 0, as illustrated by the circles in yellow. Cell surface area for each EC is shown in yellow. Junctional linearity index is calculated by dividing the length between the vertices (total length yellow lines) by the perimeter of the EC (shown in white after IF staining of VE-cadherin or β-catenin). Number of vertices is determined counting the number of vertices (contact points between cells).


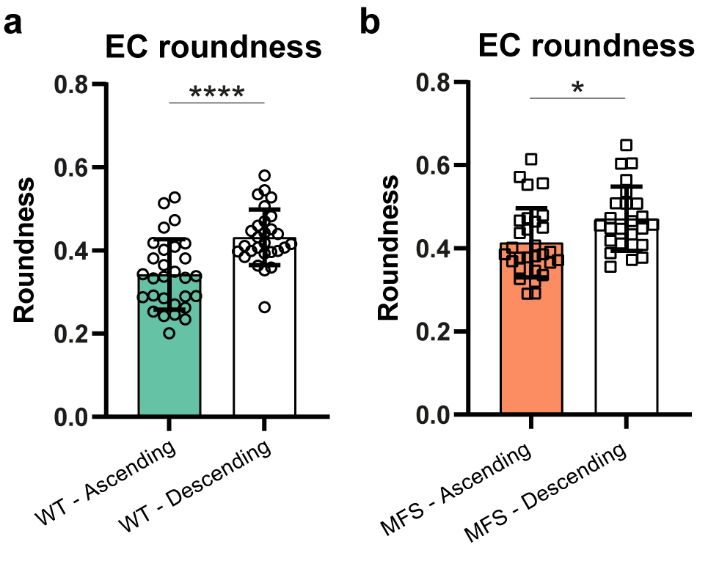


**Supplementary figure 2. Roundness of ECs in the ascending aorta versus the descending aorta.**

Bar plot comparing the roundness of ECs in the ascending and descending aorta of **(A)** WT and **(B)** *Fbn1*^C1041G/+^ MFS mice both with the age of 9 weeks. WT ascending (N=6; M/F= 3/3) n=25 images (total 424 ECs), WT descending (N=7; M/F= 3/4) n=28 images (total 420 ECs). MFS ascending (N=6; M/F= 4/2) n=33 images (total 555 ECs), MFS descending (N=6; M/F= 2/4) n=24 images (total 360 ECs). Data is shown as mean±SD with indicated datapoints. *p<0.05, ****p<0.0001 analyzed by students t-test.

**
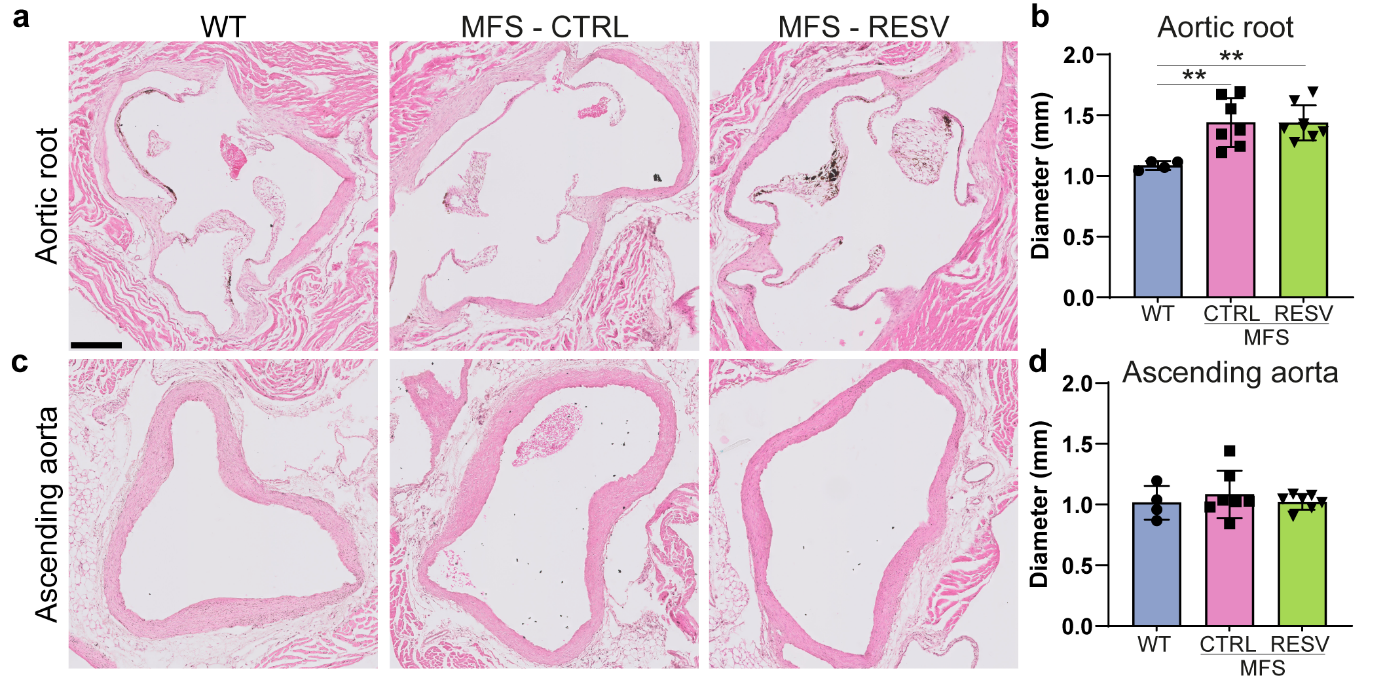
 Supplementary figure 3. Histological examination of aortic root and ascending aorta.**

**(A)** Representative histology images of the aortic root or **(C)** ascending aorta in 36 weeks old WT and Fbn1^C1041G/+^ MFS mice after HE staining. The MFS mice were treated for three weeks with either vehicle control (MFS-CTRL) or 0.1 mg/ml resveratrol (MFS-RESV). Scale bar indicates 200 µm. **(B)** Bar plot comparing the diameters of the aortic root or **(D)** ascending aorta as determined by HE stained tissue sections.


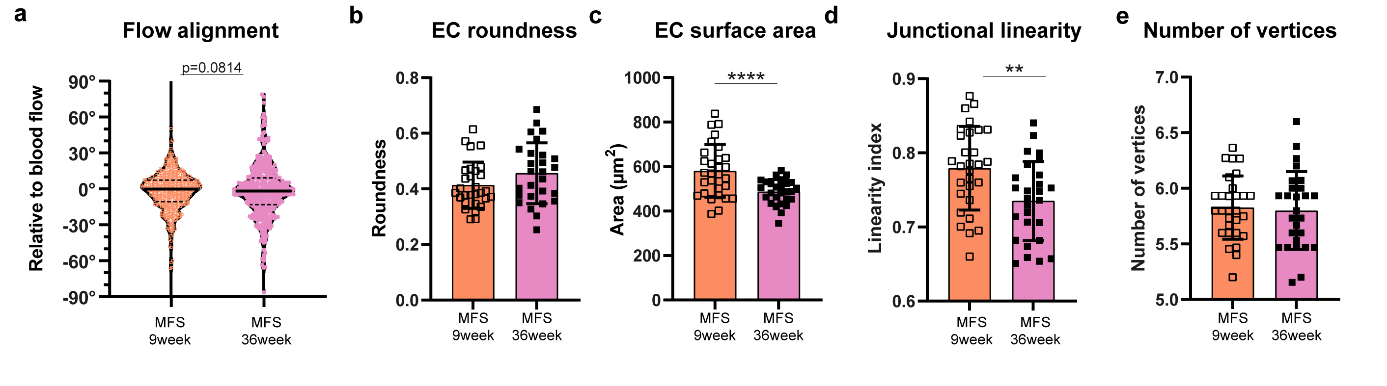


**Supplementary figure 4. Morphology of ECs in the ascending aorta of young 9 weeks old versus aged 36 weeks old MFS mice.**

**(A)** Violin plot showing quantification of EC alignment normalized to the direction of flow. Data distribution was analyzed by Kolmogorov-Smirnov test. **(B-E)** Bar plots of EC morphology parameters in the ascending aorta from 9 weeks and 36 weeks old *Fbn1*^C1041G/+^ MFS mice. All data are represented as mean±SD with indicated datapoints for MFS 9 weeks old (N=6; M/F= 4/2) n=33 images (total 555 ECs) and MFS 36 weeks old (N=6; M/F 0/6) n=24 images (total 360 ECs). **p<0.01, ****p<0.0001 analyzed by students t-test.

**
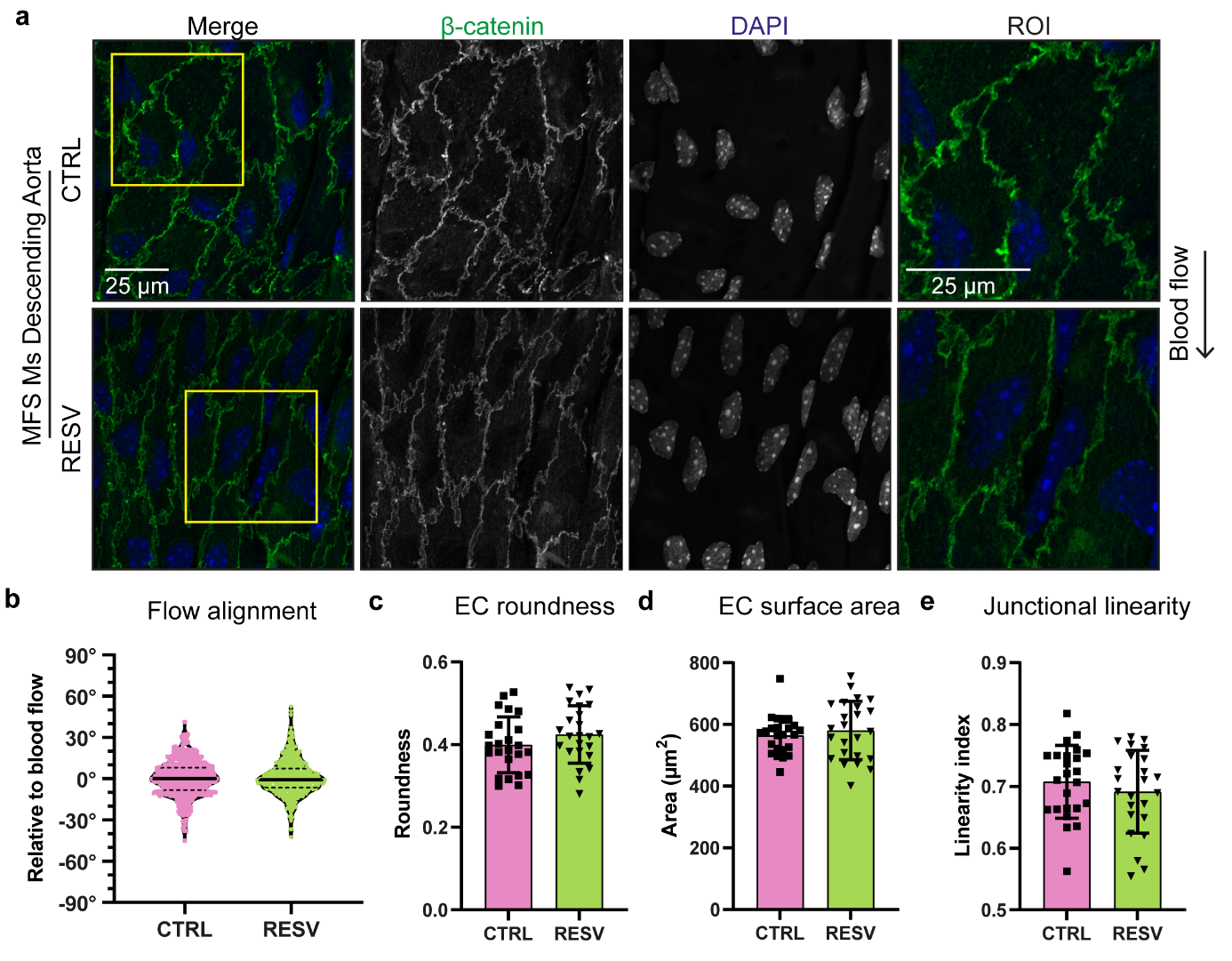
Supplementary figure 5. Resveratrol treatment induced no effect on EC shape in the descending aorta.**

**(A)** Representative *en face* IF confocal images showing β-catenin (green), and DAPI (blue) of descending aorta from 9 months old *Fbn1*^C1041G/+^ MFS mice treated for 3 weeks with RESV or vehicle. Region of interest (ROI) highlights EC junctional linearity. Arrow indicates the direction of blood flow. Scale bar: 25 μm. **B)** Violin plot showing quantification of EC alignment normalized to the direction of flow. Data distribution was analyzed by Kolmogorov-Smirnov test. **(C-E)** Bar plots of EC morphology parameters in the descending aorta of *Fbn1*^C1041G/+^ MFS mice treated for 3 weeks with vehicle control or RESV. All data are represented as mean±SD with indicated datapoints for control treatment (N=6; M/F 0/6) n=24 images (total 360 ECs) and RESV treatment (N=6; M/F 0/6) n=24 images (total 360 ECs).

**Supplementary movie 1.** Three-dimensional volumetric confocal fluorescence movie of WT descending aorta. Representative *en face* IF confocal projection showing β-catenin (green), F-actin (Red) and DAPI (blue) of descending aorta from 9 weeks old WT mice.

**Supplementary movie 2.** Three-dimensional volumetric confocal fluorescence movie of MFS descending aorta. Representative *en face* IF confocal projection showing β-catenin (green), F-actin (Red) and DAPI (blue) of descending aorta from 9 weeks old MFS mice.
